# Mapping the long-term delayed recall-based cortex-hippocampus network constrained by the structural and functional connectome: a case-control multimodal MRI study

**DOI:** 10.1186/s13195-023-01197-7

**Published:** 2023-03-24

**Authors:** Jie Ma, Mou-Xiong Zheng, Jia-Jia Wu, Xiang-Xin Xing, Yun-Ting Xiang, Dong Wei, Xin Xue, Han Zhang, Xu-Yun Hua, Qi-Hao Guo, Jian-Guang Xu

**Affiliations:** 1 Center of Rehabilitation Medicine, Yueyang Hospital of Integrated Traditional Chinese and Western Medicine, Shanghai University of Traditional Chinese Medicine, Shanghai, 200437 China; 2 School of Rehabilitation Science, Shanghai University of Traditional Chinese Medicine, Shanghai, 201203 China; 3 Department of Traumatology and Orthopedics, Yueyang Hospital of Integrated Traditional Chinese and Western Medicine, Shanghai University of Traditional Chinese Medicine, Shanghai, 200437 China; 4 School of Biomedical Engineering, ShanghaiTech University, Shanghai, 201210 China; 5 Department of Gerontology, Shanghai Jiao Tong University Affiliated Sixth People’s Hospital, Shanghai, 200233 China; 6 Engineering Research Center of Traditional Chinese Medicine Intelligent Rehabilitation, Ministry of Education, Shanghai, 201203 China

**Keywords:** Amnestic mild cognitive impairment, Multi-modal MRI, Cortical-hippocampal network, Long-term delayed recall, Connectome

## Abstract

**Background:**

Connectome mapping may reveal new treatment targets for patients with neurological and psychiatric diseases. However, the long-term delayed recall based-network with structural and functional connectome is still largely unknown. Our objectives were to (1) identify the long-term delayed recall-based cortex-hippocampus network with structural and functional connectome and (2) investigate its relationships with various cognitive functions, age, and activities of daily living.

**Methods:**

This case-control study enrolled 131 subjects (73 amnestic mild cognitive impairment [aMCI] patients and 58 age- and education-matched healthy controls [HCs]). All subjects completed a neuropsychological battery, activities of daily living assessment, and multimodal magnetic resonance imaging. Nodes of the cortical-hippocampal network related to long-term delayed recall were identified by probabilistic fiber tracking and functional connectivity (FC) analysis. Then, the main and interaction effects of the network on cognitive functions were assessed by a generalized linear model. Finally, the moderating effects of the network on the relationships between long-term delayed recall and clinical features were analyzed by multiple regression and Hayes’ bootstrap method. All the effects of cortex-hippocampus network were analyzed at the connectivity and network levels.

**Results:**

The result of a generalized linear model showed that the bilateral hippocampus, left dorsolateral superior frontal gyrus, right supplementary motor area, left lingual gyrus, left superior occipital gyrus, left superior parietal gyrus, left precuneus, and right temporal pole (superior temporal gyrus) are the left and right cortex-hippocampus network nodes related to long-term delayed recall (*P* < 0.05). Significant interaction effects were found between the Auditory Verbal Learning Test Part 5 (AVLT 5) scores and global properties of the left cortex-hippocampus network [hierarchy, clustering coefficient, characteristic path length, global efficiency, local efficiency, Sigma and synchronization (*P* < 0.05 Bonferroni corrected)]. Significant interaction effects were found between the general cognitive function/executive function/language and global properties of the left cortex-hippocampus network [Sigma and synchronization (*P* < 0.05 Bonferroni corrected)].

**Conclusion:**

This study introduces a novel symptom-based network and describes relationships among cognitive functions, brain function, and age. The cortex–hippocampus network constrained by the structural and functional connectome is closely related to long-term delayed recall.

**Supplementary Information:**

The online version contains supplementary material available at 10.1186/s13195-023-01197-7.

## Background

Amnestic mild cognitive impairment (aMCI) represents a transitionary state between normal cognitive aging and dementia and is the major risk factor for clinical dementia [1]. Early treatment of aMCI can not only maintain (or even improve) cognitive function but also reduces the likelihood of clinical dementia, which in turn should reduce the heavy mental and economic burden placed on society and families. Therefore, identifying effective treatments has scientific and clinical significance. Current medications are unsatisfactory in terms of attenuating the progression of aMCI [2]. Moreover, the etiology and pathogenesis of aMCI are not fully understood, which complicates the development of new medications [3]. Given the increasing prevalence of aMCI and lack of effective pharmacotherapies, neurorehabilitation specialists have been working toward improving symptoms using neuromodulation techniques such as transcranial magnetic stimulation (TMS). A variety of patterns have been developed and applied in clinical practice to enhance the effectiveness of TMS [4]. A meta-analysis of non-invasive brain stimulation reported that high-frequency repetitive TMS improves global cognition, and patients with Alzheimer’s disease (AD) may be more responsive than those with MCI [5]. Traditional “single-target” stimulation modulates the excitability of local brain regions (points); this may have limited the efficacy of TMS treatment, because neural connectivity (lines) and network (planes) are more important for memory function [6–8]. In addition, although aMCI is closely related to AD, they differ in clinical presentation and severity, and AD often manifests as overall cognitive decline not only in the domain of memory [9]. Therefore, it is imperative to develop a neuromodulation protocol for aMCI.

We propose a new neuromodulation target based on neural plasticity at the “line-to-plane” level, for use instead of single-target stimulation. The regulatory mechanism of our protocol is more consistent with neurophysiological mechanisms than traditional “point” modulation protocols. Specifically, we developed a novel network construction technique to identify targets for relieving the symptoms of aMCI. The core characteristic of aMCI is episodic memory impairment that does not affect daily life [10]. Delayed recall tasks, which are used for episodic memory assessment, are the most sensitive predictors of the development of aMCI [11]. A significant decline in long-term delayed recall was observed in both single- and multiple-domain aMCI [12]. Delayed recall is affected by the functions of, and connections among, multiple brain regions. In particular, the hippocampus is a major component of the limbic system implicated in long-term delayed recall performance [13]. Evidence from resting-state functional magnetic resonance imaging (rs-fMRI) of associations among reorganized/disrupted network functions, cognitive performance, and aMCI progression is growing. Changes of functional connectivity (FC) in hippocampus-related networks may be a key factor in the episodic memory impairments seen in aMCI and significantly correlate with aMCI progression [14]. The role of the cortex-hippocampus network in delayed recall has been explored at the functional level. Brueggen, K et al. reported that the decreased FC between the hippocampus and cortical brain regions may contribute to primacy recall performance in aMCI [15]. Based on the above evidence, we believe that it may be feasible to identify new treatment targets for aMCI at the line-to-plane level.

Connectome mapping may be a good way to explore the long-term delayed recall based cortex-hippocampus network, and is commonly used for identifying new treatment targets for patients with complex neurologic and psychiatric symptoms [16]. With the introduction of the concept of the “human connectome,” awareness that different brain regions causing the same symptoms may exist in a common network has grown. Recently, four consecutive articles [17–20] published in Science also verified that “connection” is essential for cognition and behavior. After the occurrence of diseases, functional or structural disorders may also occur in the cerebral circuits, and DTI imaging is considered to be a non-invasive visualization tool suitable for studying brain connectivity. These arguments provide strong support for us to find neural regulatory targets based on connectivity. There are many types of connections between brain regions, which can be broadly divided into FC and structural connectivity. Usually, the nodes of functional networks are defined in terms of functional activation or functional connections [21]. Only FC, and not structure, is considered during the construction of most functional networks. The use of previous methods may result in redundant information in the brain network that does not reflect actual brain network function. Correlating brain regions that in fact lack connections may have little impact on brain network function [22]. More notably, white matter hyperintensity is significantly associated with episodic memory dysfunction and has consequences for cognition [23]. We devised a novel network construction method, and combined the structural and functional connectome, to identify new targets for treating the symptoms of aMCI. The main advantage of our novel network construction method is that it may eliminate the influence of irrelevant signals, thus allowing for greater focus on the symptoms of interest and precision treatment.

To explore the role of the cortex-hippocampus network in long-term delayed recall, and to investigate its relationships with various other cognitive functions at the connectivity and network levels, aMCI patients and age- and education-matched healthy controls (HCs) were recruited and assessed via a neuropsychological battery, multimodal magnetic resonance imaging (MRI), and two activities of daily living questionnaire. This study not only introduces a novel symptom-based network but also describes the underlying relationships among cognitive functions, brain function and brain structure.

## Materials and methods

### Participants

The 131 subjects in this case-control study (73 aMCI patients and 58 age- and education-matched HCs) were recruited from January 2020 to July 2022 in Shanghai, China. The study was approved by the ethics committee of Shanghai Jiao Tong University Affiliated Sixth People’s Hospital (institutional review board number: 2019-041), and all participants provided written informed consent before any procedures were performed. All subjects underwent the neuropsychological evaluation and MRI before any treatment aimed at improving cognitive function.

The inclusion criteria for the aMCI group were as follows: (1) a diagnosis of aMCI [24], i.e., Mini-Mental State Examination (MMSE) score > 24, Auditory Verbal Learning Test part 5 (pertaining to long-term delayed recall; hereafter, AVLT 5) score ≥ 1 standard deviation (SD) below the norm according to education level and age, Activities of Daily Living Scale (ADL; Chinese version) ≤ 1 item, and absence of dementia according to the National Institute on Aging-Alzheimer’s Association (NIA-AA) criteria [25]; a (2) Clinical Dementia Rating (CDR) [26] memory score of 0.5; (3) cognitive complaints for ≤ 1 year; (4) no previous treatment for improving cognitive function.

The inclusion criteria for the HC group were as follows: (1) no cognitive complaints; (2) a neuropsychological evaluation score below the threshold for impairment; (3) a CDR score of 0; (4) an ADL-Chinese version = 0 item.

The exclusion criteria for all individuals were as follows: (1) a diagnosis of any type of dementia; (2) cerebral parenchymal lesions on brain MRI (e.g., malacia or tumor); (3) history of other neurological disease, or psychiatric disease; (4) severe cardiac, hepatic or renal dysfunction; (5) severe hearing or visual impairment; (6) unsuitable for MRI; and (7) < 6 years of education.

### Neuropsychological evaluation

Two senior neuropsychologists with > 10 years of work experience performed the neuropsychological evaluation without knowledge of the clinical diagnosis; another senior neuropsychologist then reviewed the assessment results. All individuals completed a neuropsychological battery. General cognitive function was evaluated by the MMSE [27], Montreal Cognitive Assessment-Basic (MoCA-B) [28], and Addenbrooke’s Cognitive Examination III (ACE-III) [29]. Memory function was evaluated by the AVLT [30]. Language function was assessed by the Boston Naming Test (BNT) [31] and Animal Verbal Fluency Test (AFT) [32]. Executive function was evaluated by the Shape Trail Test (STT) [33] and Stroop Test [34]. Attention was assessed by the Symbol Digit Modalities Test (SDMT) [35] and Digit Span Test (DST) [36]. Spatial function was evaluated by the Judgment of Line Orientation (JLO) test [37]. Performance in activities of daily life was assessed by the Everyday Cognition (ECog) scale [38] and Pittsburgh Sleep Quality Index (PSQI) [39]. Physical performance was evaluated by the Timed Up and Go Test (TUGT) [40]. Emotional state was assessed by the Hamilton Depression Scale (HAMD) and Hamilton Anxiety Scale (HAMA) [41].

### Image acquisition

MRI data were acquired using a MAGNETOM Prisma 3.0-Tesla scanner (Siemens, Erlangen, Germany). The participants were instructed to close their eyes but not fall asleep and remain calm; their heads were immobilized with foam pads, and their ears were plugged with earplugs. Light and sound shielding were applied before and during the scan. Resting-state fMRI data were obtained with a gradient-recalled echo-planar imaging (EPI) sequence with the following parameters: transverse plane; repetition time (TR), 800 ms; echo time (TE), 37 ms; field of view (FOV), 208 × 208 mm^2^; matrix size, 104 × 104; flip angle, 52°; slice thickness, 2.0 mm; slice number, 72; gap, 0 (voxel size = 2.0 × 2.0 × 2.0 mm^3^); and number of acquisitions, 488. A T1-weighted magnetization-prepared rapid acquisition gradient echo scan was then performed, with the following parameters: sagittal acquisition; matrix size, 320 × 320; FOV, 256 × 256 mm^2^; slice thickness, 0.8 mm; voxel size, 0.8 × 0.8 × 0.8 mm^3^; TR, 3000 ms; TE, 2.56 ms; inversion time, 1100 ms; flip angle, 7°; slice number, 208. Diffusion tensor imaging (DTI) was performed using a single-shot spin EPI in the axial plane: TR, 10,000 ms; TE, 89 ms; flip angle, 90°; slice thickness, 2.0 mm; in-plane resolution, 1.875 mm; 60 non-colinear directions (b, 1000 s/mm^2^), and two b0 images.

### Imaging data preprocessing and processing

#### T1-weighted image analysis

Preprocessing and voxel-based morphometry were performed using the Computational Anatomy Toolbox (CAT12; http://www.neuro.uni-jena.de/cat/), which was run through SPM12 (http://www.fil.ion.ucl.ac.uk/spm) and MATLAB (version 2013b; MathWorks, Natick, MA, USA). All images were processed using the default parameters of the toolboxes. Data preprocessing and processing included the following steps: (1) edges were cropped to leave only the brain; (2) spatial registration of images to a reference brain space template (the International Consortium for Brain Mapping East Asian brain template) was performed via DARTEL registration; (3) images were segmented into gray matter (GM), white matter, and cerebrospinal fluid; (4) bias correction of intensity non-uniformities across images was performed; (5) linear registration and nonlinear modulation was achieved by scaling according to volume changes induced by spatial registration; and (6) the total intracranial volume, GM, white matter, and hippocampal volume were extracted for each subject. Image quality was checked carefully after each step.

#### Cortical-hippocampal network definition

**Fig. 1:**
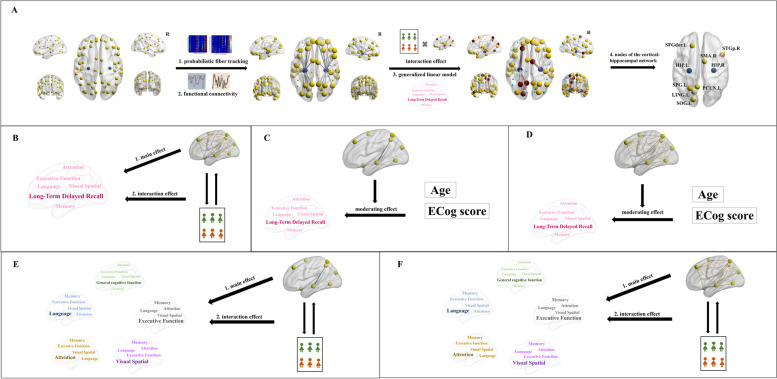
Flow chart of the study. **A** The process through which the cortical-hippocampal network was defined in the context of long-term delayed recall. **B** Main and interaction effects of the global properties of the left cortical-hippocampal network on long-term delayed recall. **C** Moderating effects of the FC of the cortical-hippocampal network on the relationships of the AVLT 5 score with age and the ECog score. **D** Moderating effects of the global properties the left cortical-hippocampal network on the relationships of the AVLT 5 score with age and the ECog score. **E** Associations of the FC of the cortical-hippocampal network with other cognitive functions. **F** Associations of the global properties of the left cortical-hippocampal network with other cognitive functions. AVLT 5, Auditory Verbal Learning Test part 5 (long-term delayed recall); FC, functional connectivity

As shown in Fig. 1A, identification of the cortical-hippocampal network involved four steps. First, cortical nodes with white matter fiber connections were identified by probabilistic fiber tracking, on the basis of two voxel-based seeds (left and right hippocampus) and two voxel-based target maps (left and right cortical areas). Then, FCs between cortical nodes and the hippocampus were calculated. Third, a generalized linear model was constructed to analyze the relationship between long-term delayed recall and FCs; group × FCs interaction effect was analyzed. Fourth, nodes of the left and right cortical-hippocampal network were identified. Probabilistic fiber tracking and FC were performed as follows.

#### Analysis of probabilistic fiber tracking

Nodes of the cortical-hippocampal network were identified based on probabilistic fiber tracking of subject-specific native space using FMRIB Software Library (FSL) v5.0 (http://www.fmrib.ox.ac.uk/). The DTI data of the HC group were preprocessed and processed as follows [42]. (1) Preprocessing: datasets were corrected for eddy currents and head motion, nonbrain tissue was stripped, noise was reduced, diffusion tensors were fitted to each voxel, and voxel-wise fractional anisotropy (FA) maps were calculated for each participant. (2) Seed and target areas preparation: seeds and target maps were created using an automated anatomical labeling template [43], including two voxel-based seeds (left and right hippocampus) and two voxel-based target maps (left and right cortical areas) (Table 1). All seed and target areas acquired in the standard atlas were converted to subject-specific native space. We registered FA maps to the corresponding T1-weighted scans in native space using FMRIB’s Linear Image Registration Tool (FLIRT) and nonlinearly transformed to register the T1-weighted images to standard MNI space. Subsequently, the transformation parameters estimated above were inversed and then applied to the seed and cortical areas in standard space. (3) Probabilistic tracking: fiber tracking was initiated using all voxels within the seed mask in diffusion space, to generate 5000 streamline samples with a step length of 0.5 mm, curvature threshold of 0.2, and maximum of 2000 steps. A target mask (left or right cortical area) was used, and the distribution of fiber orientations was calculated using the left or right hippocampus as a seed mask. (4) Connection identification: we used FSL to identify the voxel with the maximum connectivity value within the connectivity distribution map of each participant and identified a threshold of 15% (relative to the maximum connectivity value) as the optimum threshold value. After probabilistic fiber tracking, the node with white matter fiber connection to the hippocampus were identified (Table 1).

**Table 1:** The seeds and target brain regions by using the automated anatomical labeling (AAL) template

The seeds and targets (left and right)	Abbreviation	With WM connection	Final brain areas
Precentral gyrus	PreCG		
Superior frontal gyrus, dorsolateral part	SFGdor	L, R	L
Superior frontal gyrus, orbital part	SFGorb	L, R	
Middle frontal gyrus	MFG		
Middle frontal gyrus, orbital part	MFGorb	R	
Inferior frontal gyrus, opercular part	IFGoper	R	
Inferior frontal gyrus, triangular part	IFGtri	R	
Inferior frontal gyrus, orbital part	IFGorb	L, R	
Rolandic operculum	ROL	L	
Supplementary motor area	SMA	L, R	R
Superior frontal gyrus, medial part	SFGmed	R	
Superior frontal gyrus, medial orbital part	SFGmedorb	L, R	
Hippocampus	HIP	L, R	L, R
Cuneus	CUN		
Lingual gyrus	LING	L, R	L
Superior occipital gyrus	SOG	L, R	L
Middle occipital gyrus	MOG	L	
Inferior occipital gyrus	IOG		
Postcentral gyrus	PoCG	R	
Superior parietal gyrus	SPG	L, R	L
Inferior parietal gyrus	IPL		
Supramarginal gyrus	SMG		
Angular gyrus	ANG	R	
Precuneus	PCUN	L, R	L
Paracentral lobule	PCL		
Heschl gyrus	HES	L	
Superior temporal gyrus	STG	L, R	
Superior temporal gyrus, temporal pole	STGp	L, R	R
Middle temporal gyrus	MTG	L, R	
Middle temporal gyrus, temporal pole	MTGp	L, R	
Inferior temporal gyrus	ITG	L, R	

*WM* white matter, *L* left, *R* right

#### Functional image analysis

##### Data preprocessing

Functional images from each participant were preprocessed using the RESTPlus software [44] and SPM12, which were run in the MATLAB environment (version 2013b; MathWorks, Natick, MA, USA). The blood oxygenation level-dependent (BOLD) signals in the aMCI and HC groups were preprocessed as follows. The first 10 volumes of each fMRI scan were discarded, and slice timing, motion correction (for excessive translation or rotation, i.e., ≥ 3 mm and 3°, respectively), spatial normalization with DARTEL registration, spatial smoothing (using a 6 × 6 × 6 mm^3^ Gaussian kernel; full-width at half-maximum = 6 mm), regression of nuisance variables (white matter, cerebrospinal fluid, and six head motion parameters), and linear trend removal were performed.

##### FC analysis

Functional images from each participant were processed using the RESTPlus software [44] and SPM12, which were run in the MATLAB environment (version 2013b; MathWorks, Natick, MA, USA). (1) Region of interest (ROI) extraction: The 39 nodes in Table 1 defined the ROI used for “seed-based ROI-wise” FC analysis. (2) Pearson correlation analyses of the mean time series between all pairs of nodes were performed and the coefficients were standardized (via Fisher’s *Z* transform).

##### Functional network analysis

Functional images for each participant were processed using the GRETNA toolbox [45] and SPM12, which were run in the MATLAB environment (version 2013b). (1) FC matrix construction: The FC matrix was constructed within the long-term delayed recall-based left cortical-hippocampal network (Table 1). (2) Graph matrix computation [46]: to obtain networks with the same number of edges, network metrics were calculated using a preselected sparsity value (range: 0.15–0.45) [47] to generate a set of undirected and unweighted binarized networks. Network density was adjusted for adjacent matrices with an increment of 0.01. (3) We analyzed eight global properties: assortativity, hierarchy, synchronization, Sigma, the clustering coefficient (Cp), characteristic path length (Lp), global efficiency (Eglobal), and local efficiency (Elocal) (see Supplemental Table 1 for definitions). For the functional network analyses, the area under the curve (AUC) over the sparsity range for each global metric was calculated to provide a scalar for statistical comparison.

### Statistical analysis

#### Group comparison of clinical characteristics

The SPSS 26.0 software (IBM Corp., Armonk, NY, USA) was applied for the statistical analysis. A two-sample *t*-test and the *χ*^2^ test were used for intergroup comparisons of continuous and categorical variables, respectively. Cohen’s *d* was calculated as a measure of effect size. Two-tailed *P*-values < 0.05 were considered significant.

#### Relationship between long-term delayed recall and clinical features

To determine the contributions of clinical features (age, years of education, PSQI, ECog score, and TUGT time) to AVLT 5 scores, a generalized linear model was constructed. Main effects of the clinical features on the AVLT 5 scores (the dependent variable) were tested, along with interaction effects. Pearson’s correlation was used to analyze the relationships between AVLT 5 scores and those in other cognitive domains. The Bonferroni correction was used for multiple comparison correction, and two-tailed *P*-values < 0.05 were considered significant.

#### Associations of the functional connectivity of the cortical-hippocampal network with cognitive performance

To determine the main and interaction effects of the FCs of the cortical-hippocampal network on cognitive performance (dependent variable), we constructed two generalized linear models (GLMs) with cognitive score as the dependent variable. We put ipsilateral FCs in one model to explain the interrelationships and interactions between the variables, and group × FCs as interactive variable (Fig. 1B, E, F).

#### Associations of the global property of the left cortical-hippocampal network with cognitive performance

To determine the main and interaction effects of the global property of the left cortical-hippocampal network on cognitive performance (dependent variable), a generalized linear model was constructed with cognitive score as the dependent variable, the global property of the left cortical-hippocampal network as independent variable separately, and group × global property for interactive variable (Fig. 1B, E, F). The Bonferroni correction was used for multiple comparison correction.

#### Moderating effect of the left cortical-hippocampal network

The moderating effects of the FC and global properties of the left cortical-hippocampal network on the relationships of the AVLT 5 score with age and the ECog score were analyzed using multiple regression and Hayes’ bootstrapping method (using the PROCESS macro and 5000 bootstrap samples). AVLT 5 score is the dependent variable, age/ECog score is the independent variable, and FC/global properties of the cortical-hippocampal network is the moderator (Fig. 1C, D).

## Results

### Clinical characteristics

#### Group comparison

The clinical characteristics of the participants are summarized in Table 2. The aMCI and HC groups differed significantly in cognitive performance and performance of activities of daily life (*P* < 0.05), but not in baseline characteristics, physical performance, emotional state, or brain volume (*P* > 0.05).

**Table 2:** Comparison of clinical characteristics between amnestic mild cognitive impairment (aMCI) and healthy control (HC) groups

Characteristics	HC ***n*** = 58	aMCI ***n*** = 73	***P***-value	Cohen’s ***d*** (95%CI)
**Basic characteristics**				
Gender (% female)	42 (72.4%)	43 (58.9%)	0.141	--
Age (y)	64.64 (6.87)	66.55 (6.97)	0.119	--
Education (y)	10.69 (3.16)	10.34 (2.74)	0.501	--
Smoking (%)	4 (6.98%)	11 (15.07%)	0.175	--
Drinking (%)	7 (12.07%)	4 (5.48%)	0.214	--
Height (cm)	178.49 (109.82)	174.56 (998.30)	0.830	--
Weight (kg)	61.73 (9.50)	63.24 (10.91)	0.404	--
Hypertension	25 (43.10%)	23 (31.51%)	0.203	--
Hypercholesterolemia	6 (10.34%)	8 (10.96%)	1.000	--
Diabetes	11 (18.97%)	14 (19.18%)	1.000	--
**Daily life performance**				
ECog	17.41 (4.98)	21.41 (7.42)	**< 0.001**	0.647 (0.307, 0.997)
PSQI	5.85 (2.87)	6.86 (4.83)	0.158	0.25 (0.10, 0.60)
**Physical performance**				
TUGT_time (s)	9.42 (1.76)	9.89 (2.30)	0.191	0.23 (0.12, 0.57)
**Emotional performance**				
HAMD	2.12 (2.52)	1.60 (2.05)	0.197	0.23 (0.12, 0.58)
HAMA	2.69 (2.29)	2.88 (2.34)	0.647	0.08 (0.27, 0.43)
**Cognitive performance**				
**General cognitive function**				
MMSE	28.33 (1.43)	26.70 (1.71)	**< 0.001**	1.024 (0.656, 1.389)
MoCA_B	26.36 (1.91)	22.62 (3.15)	**< 0.001**	1.478 (1.084, 1.867)
ACE_III	82.62 (7.15)	77.07 (7.09)	**< 0.001**	0.780 (0.421, 1.137)
**Memory function**				
AVLT	33.38 (7.11)	17.55 (5.14)	**< 0.001**	2.508 (2.016, 2.994)
AVLT_N1	4.33 (1.33)	2.89 (1.02)	**< 0.001**	1.195 (0.811, 1.573)
AVLT_N2	6.98 (1.85)	4.55 (1.23)	**< 0.001**	1.519 (1.109, 1.923)
AVLT_N3	8.38 (1.70)	5.34 (1.59)	**< 0.001**	1.849 (1.434, 2.258)
AVLT_N4	6.98 (1.82)	2.70 (1.60)	**< 0.001**	2.522 (2.057, 2.981)
AVLT_N5	6.71 (1.88)	2.07 (1.47)	**< 0.001**	2.790 (2.303, 3.271)
AVLT_N6	6.47 (2.46)	2.27 (1.61)	**< 0.001**	1.973 (1.524, 2.415)
AVLT_N7	22.05 (2.02)	18.30 (2.38)	**< 0.001**	1.684 (1.280, 2.082)
**Language function**				
BNT	24.64 (2.42)	21.38 (3.83)	**< 0.001**	1.042 (0.672, 1.409)
AFT	18.40 (3.51)	15.84 (3.80)	**< 0.001**	0.697 (0.341, 1.051)
**Executive function**				
STT_B (s)	117.17 (35.91)	175.38 (78.30)	**< 0.001**	0.988 (0.621, 1.354)
Stroop test_A	24.00 (0)	23.95 (0.23)	0.071	0.29 (0.06, 0.64)
Stroop test_B	23.03 (1.97)	22.18 (2.56)	**0.038**	0.381 (0.032, 0.728)
**Attention function**				
SDMT_correct	43.79 (9.85)	28.34 (12.50)	**< 0.001**	1.391 (1.005, 1.774)
DST_seq	7.40 (1.38)	7.07 (1.41)	0.183	0.24 (0.11, 0.58)
DST_rev	4.85 (1.20)	4.45 (1.12)	0.055	0.35 (0, 0.69)
**Spatial function**				
JLO	21.75 (3.70)	21.32 (4.05)	0.526	0.11 (0.24, 0.46)
**Brain volumes (cm**^**3**^**)**				
TIV	1362.29 (154.73)	1366.16 (126.68)	0.88	0.03 (0.32, 0.38)
GM	589.90 (53.05)	583.61 (50.86)	0.49	0.12 (0.23, 0.47)
WM	485.93 (64.86)	478.46 (58.63)	0.49	0.12 (0.23, 0.47)
Left hippocampus	2.90 (0.27)	2.84 (0.40)	0.31	0.17 (0.18, 0.52)
Right hippocampus	3.18 (0.31)	3.13 (0.38)	0.40	0.14 (0.21, 0.49)

Data are mean (standard deviation) or number of participants in each group (% of total). *Abbreviations*: *ACE-III*, Addenbrooke’s Cognitive Examination; *aMCI*, amnestic mild cognitive impairment; *AFT*, Animal Verbal Fluency Test; *AVLT_N1*, Auditory Verbal Learning Test, First Immediate Recall; *AVLT_N2*, Auditory Verbal Learning Test, Second Immediate Recall; *AVLT_N3*, Auditory Verbal Learning Test, the Third Immediate Recall; *AVLT_N4*, Auditory Verbal Learning Test, Short-Term Delay Recall; *AVLT_N5*, Auditory Verbal Learning Test, Long-Term Delay Recall; *AVLT_N6*, Auditory Verbal Learning Test, Long Delay Cued Recall; *AVLT_N7*, Auditory Verbal Learning Test, Recognition; *BNT*, Boston Naming Test; *CI*, confidence interval; *DST_rev*, Digit Span Test, reverse; *DST_seq*, Digit Span Test, sequence; *ECog*, Everyday Cognition; *GM*, gray matter; *HAMA*, Hamilton Anxiety Scale; *HAMD*, Hamilton Depression Scale; *HC*, healthy control; *JLO*, Judgment of Line Orientation; *MMSE*, Mini-Mental State Examination; *WM*, white matter; *MoCA_B*, Montreal Cognitive Assessment-basic; *PSQI*, Pittsburgh Sleep Quality Index; *SDMT_correct*, Symbol Digit Modalities Test, correct number; stt_b, Shape Trails Test, part B; *TUGT_time*, Timed Up and Go Test; *TIV*, total intracranial volumes

#### Relationships between long-term delayed recall and clinical features

**Fig. 2:**
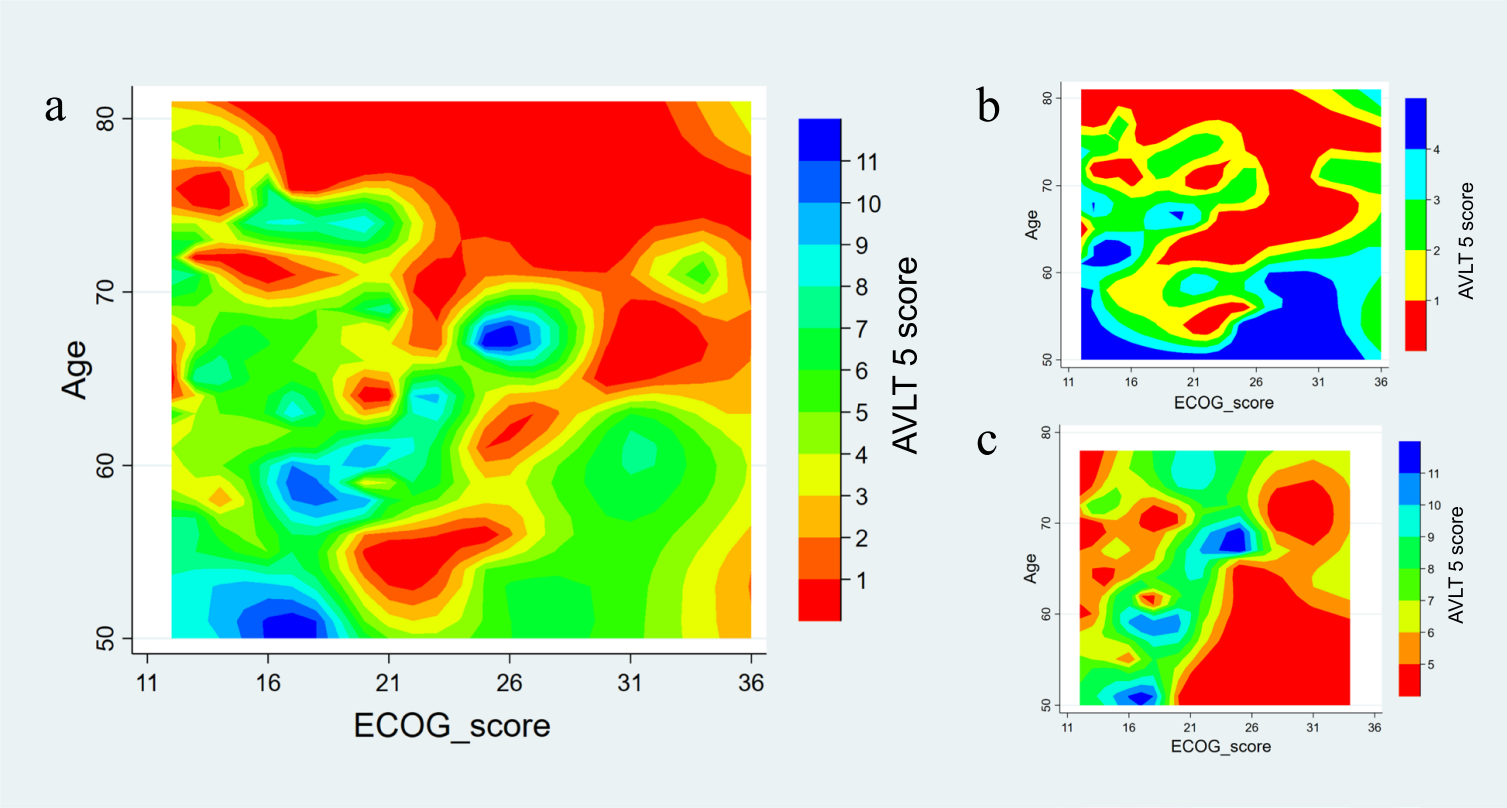
Relationships between the AVLT 5 score and clinical features. **A** Relationships among age, the ECog score and the AVLT 5 score in the entire cohort. **B** Relationships among age, the ECog score, and the AVLT 5 score in the aMCI group. **C** Relationships among age, the ECog score and the AVLT 5 score in the HC group. AVLT 5, Auditory Verbal Learning Test part 5 (long-term delayed recall); aMCI, amnestic mild cognitive impairment; HC, healthy control

**Fig. 3:**
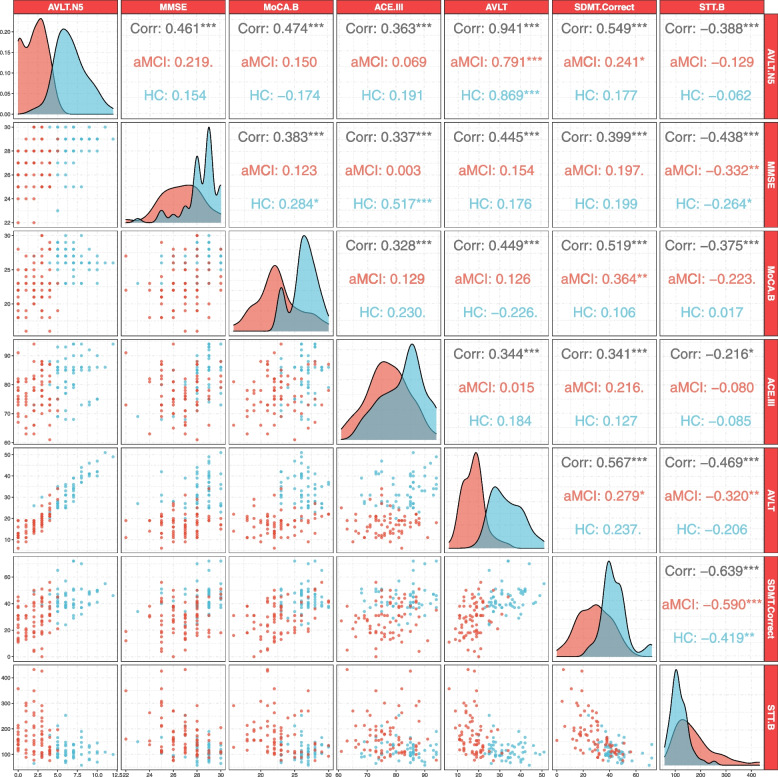
Correlations between the AVLT 5 score and scores in other cognitive domains. The correlations between the AVLT 5 score and scores in other cognitive domains were analyzed by Pearson’s correlation. Correlation coefficients between two cognitive functions are shown in the upper right triangle area. Three correlation coefficients are provided for every correlation between two cognitive functions (one each for the entire cohort, aMCI group and HC group). Scatter plots for each correlation are shown in the sitting triangle area. The nuclear density curves of each cognitive score are displayed in the diagonal cells, where blue represents the healthy control group and red represents the aMCI group. aMCI, amnestic mild cognitive impairment; HC, healthy control; AVLT, Auditory Verbal Learning Test; AVLT 5, Auditory Verbal Learning Test part 5 (long-term delayed recall); MMSE, Mini-Mental State Examination; MoCA-B, Montreal Cognitive Assessment-Basic; ACE-III, Addenbrooke’s Cognitive Examination III; SDMT, Symbol Digit Modalities Test; STT, Shape Trail Test

A generalized linear model was constructed with AVLT 5 score as the dependent variable and age, years of education, PSQI, ECog score, and the TUGT time as independent variables. Significant correlations were found between the AVLT 5 score and age (*P* < 0.001) and ECog score (*P* = 0.01). There was a significant group × age interaction effect on the AVLT 5 score (*P* < 0.001), but this was not seen for the group × ECog score interaction (*P* > 0.05) (Fig. 2). As shown in Fig. 3, there were significant correlations of the AVLT 5 score with the MMSE (*r* = 0.461, *P* < 0.05 Bonferroni corrected), MoCA-B (*r* = 0.474, *P* < 0.05 Bonferroni corrected), ACE III (*r* = 0.363, *P* < 0.05 Bonferroni corrected), AVLT (*r* = 0.941, *P* < 0.05 Bonferroni corrected), SDMT (*r* = 0.549, *P* < 0.05 Bonferroni corrected), and STT part B (hereafter, STT_B; *r* = − 0.388, *P* < 0.05 Bonferroni corrected).

### Identification of the cortical-hippocampal network associated with long-term delayed recall

In a generalized linear model, group × FC interaction effects for the AVLT 5 score were analyzed. Significant effects of FCs on the AVLT 5 score were seen in the HC group, including for FC_HIP.L-SFGdor.L_ (*B* = 3.192, *P* = 0.028), FC_HIP.L-LING.L_ (*B* = 4.238, *P* = 0.003), FC_HIP.L-SOG.L_ (*B* = 3.837, *P* = 0.049), FC_HIP.L-SPG.L_ (*B* = − 7.282, *P* = 0.002), FC_HIP.L-PCUN.L_ (*B* = 5.302, *P* = 0.017), FC_HIP.R-SMA.R_ (*B* = 6.738, *P* = 0.005), and FC_HIP.R-STGp.R_ (*B* = 4.43, *P* = 0.015). Therefore, the left hippocampus, left dorsolateral superior frontal gyrus, left lingual gyrus, left superior occipital gyrus, left superior parietal gyrus, and left precuneus were identified as the nodes in the left cortical-hippocampal network; the right hippocampus, right supplementary motor area, and right temporal pole (superior temporal gyrus) were identified as the nodes in the right cortical-hippocampal network (Fig. 1A, Table 1).

### Associations of global properties of the left cortical-hippocampal network with long-term delayed recall

Group × global property interaction effects for the AVLT 5 score showed that there were significant effects of hierarchy (*B* = − 8.361, *P* < 0.05 Bonferroni corrected), E_local_ (*B* = − 23.20, *P* < 0.05 Bonferroni corrected), Cp (*B* = − 24.379, *P* < 0.05 Bonferroni corrected), Lp (*B* = − 5.30, *P* < 0.05 Bonferroni corrected), and synchronization (*B* = − 157.53, *P* < 0.05 Bonferroni corrected) in the aMCI group; group × global property interaction effects for the AVLT 5 score showed that there were significant effects of E_globle_ (*B* = 28.956, *P* < 0.05 Bonferroni corrected), E_local_ (*B* = 13.283, *P* < 0.05 Bonferroni corrected), Cp (*B* = 13.46, *P* < 0.05 Bonferroni corrected), Sigma (*B* = 9.384, *P* < 0.05 Bonferroni corrected), and synchronization (*B* = 182.37, *P* < 0.05 Bonferroni corrected) in the HC group. The results for main effects were not significantly different (uncorrected).

### Moderating effect of the cortical-hippocampal network

#### Functional connectivity of the cortical-hippocampal network modulated the relationship between long-term delayed recall and age/ECog score

**Fig. 4:**
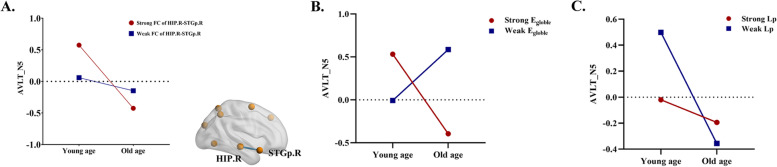
Moderating effect of the cortical-hippocampal network. **A** The relationship between AVLT_N5 score and age was reduced by FC_HIP.R-STGp.R_ (*r* = − 0.198, *P* = 0.034 uncorrected). **B** The relationship between AVLT_N5 score and age was reduced by E_globle_ (*r* = − 0.190, *P* = 0.021 uncorrected) of the left cortical-hippocampal network. **C** The relationship between AVLT_N5 score and age was increased by Lp (*r* = 0.170, *P* = 0.044 uncorrected) of the left cortical-hippocampal network. AVLT_N5, Auditory Verbal Learning Test part 5 (long-term delayed recall); FC, functional connectivity; HIP, hippocampus; STGp, temporal pole (superior temporal gyrus); R, right

The results of moderating effect showed that the relationship between AVLT 5 score and age was reduced by FC_HIP.R-STGp.R_ (*r* = − 0.198, *P* = 0.034 uncorrected) (Fig. 4A). FCs of the cortical-hippocampal network were no significant moderating effect on the relationship between AVLT 5 score and ECog score.

#### Global properties of the left cortical-hippocampal network modulated the relationship between long-term delayed recall and age/ECog score

The results of moderating effect showed that the relationship between AVLT 5 score and age was reduced by E_globle_ (*r* = − 0.190, *P* = 0.021 uncorrected) and increased by Lp (*r* = 0.170, *P* = 0.044 uncorrected) (Fig. 4B, C). Global properties of the left cortical-hippocampal network were no significant moderating effect on the relationship AVLT 5 score and ECog score.

### Effects of the cortical-hippocampal network on other cognitive functions

#### Associations between functional connectivity of the cortical-hippocampal network and other cognitive functions

**Fig. 5:**
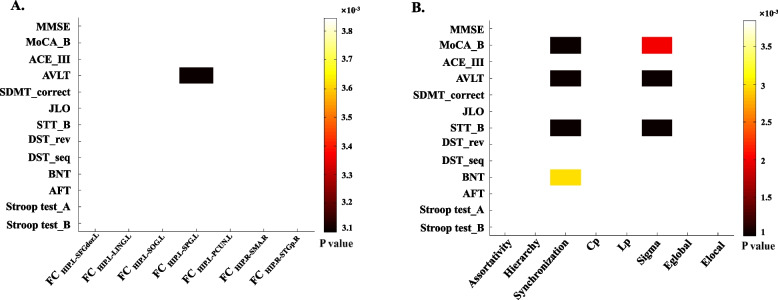
Associations of the functional connectivity and global properties of the cortical-hippocampal network with other cognitive functions. **A** Interaction effects of FC metrics of the cortical-hippocampal network on other cognitive functions. **B** Interaction effects of global properties of the left cortical-hippocampal network on other cognitive functions. All the results were passed by Bonferroni correction. ACE-III: Addenbrooke’s cognitive examination; AFT: Animal verbal fluency test; AVLT: Auditory Verbal Learning Test; BNT: Boston Naming Test; DST_rev: Digit Span Test, Reverse; DST_seq: Digit Span Test, Sequence; FC, functional connectivity. JLO, Judgment of Line Orientation; MMSE, Mini-Mental State Examination; MoCA_B, Montreal Cognitive Assessment-Basic; SDMT_correct, Symbol Digit Modalities Test, Correct Number; STT_B: Shape Trails Test, part B

Group × FC interaction effects for other cognitive functions showed that there was a significant effect of FC_HIP.L-SPG.L_ on the AVLT score in the HC group (*B* = − 17.97, *P* < 0.05 Bonferroni corrected) (Fig. 5A). The results for main effects were not significantly different (uncorrected).

#### Associations of global properties of the left cortical-hippocampal network with other cognitive functions

A generalized linear model was fitted with group × global property interaction effects for the other cognitive functions. The results are shown in Fig. 5B (*P* < 0.05 Bonferroni corrected). The results for main effects were not significantly different (uncorrected).

## Discussion

Assessment of long-term delayed recall is the current gold standard for detecting episodic memory problems, which have proven to be a consistently strong neuropsychological predictor of conversion from MCI to AD, and an early clinical manifestation of incipient AD [48, 49]. Herein, we characterized the cortical-hippocampal network, constrained by the structural and functional connectome, in the context of long-term delayed recall. Nodes of the cortical-hippocampal network include the bilateral hippocampus, left dorsolateral superior frontal gyrus, right supplementary motor area, left lingual gyrus, left superior occipital gyrus, left superior parietal gyrus, left precuneus, and right temporal pole. Interestingly, effects of FC metrics on the AVLT 5 score were mainly seen in the HC group, possibly because aMCI disrupts connections between long-term delayed recall and FC in cortical and hippocampal regions. It may be worthwhile to use TMS to potentially restore these connections and thus improve cognitive function in aMCI patients. We also showed that the FC and global properties of the identified network directly and indirectly affect multiple domains of cognition in aMCI. Our findings point to potential therapeutic targets at the line-to-plane level for improving cognition in multiple domains in aMCI.

Our exploration of the cortical–hippocampal network in the context of long-term delayed recall is consistent with findings regarding the neuroscientific basis of memory. Episodic memory involves emotional processing, i.e., processing of sense perception data, via information acquisition, storage and encoding, and extraction steps; this requires synergistic activity of different brain areas [50]. Regarding contextual memory, information acquisition often requires involvement of multiple domains, such as the visual, auditory, verbal, and attention domains. The brain stores memories in a distributed manner; for example, verbal information pertinent to situational memory is stored in verbal brain areas, while visual memory information is stored in visual brain areas. The long-term delayed recall-based cortex–hippocampus network constrained by the structural and functional connectome in our study contains the dorsolateral superior frontal gyrus, superior occipital gyrus, and superior parietal gyrus that are associated with information acquisition and storage. Long-term memory encoding is age-related, and there is a correlation between hippocampal activity [51] and dorsolateral superior frontal gyrus/superior parietal gyrus during memory encoding in older adults [52], which are also the core brain area of our network. In some fMRI study of memory consolidation, healthy subjects showed significantly enhanced functional connectivity between the hippocampus and temporal/occipital lobe when performing a long-delay recall task [53, 54], which is similar to our findings. In the process of information retrieval/extraction, the precuneus and angular gyrus are associated with temporal retrieval, the dorsolateral superior frontal gyrus and parietal gyrus are involved in spatial retrieval, and the medial anterior temporal area is activated in object-related retrieval or scene recognition [55, 56]. Among them, precuneus, dorsolateral superior frontal gyrus, parietal, and temporal gyrus are consistent with our findings. In our study, the nodes of the cortical–hippocampal network correspond to brain regions are involved in the process of information acquisition, storage, encoding, and extraction steps. We showed that the network is constrained by the structural and functional connectome, which is important for identifying therapeutic targets for aMCI symptoms.

Specifically, the prefrontal cortex (especially the left prefrontal cortex) is involved in situational memory formation and is thought to encode a key regulator of hippocampal activation and long-term memory formation [57]. Memory is a selective process; a large amount of information is stored in long-term memory, where the activation of irrelevant memory traces may interfere with retrieval. Thus, retrieval requires not only activation of relevant memory traces, but also some degree of inhibitory control over interfering memories. The prefrontal cortex exerts top-down control over information processing throughout the brain, which plays a key role in preventing interference [58, 59]. Attention is critical for episodic memory. Dorsal areas involved in attention, including the superior parietal lobules, precuneus, and supplementary motor cortex, allocate resources for top-down attention during cue retrieval [60]. The occipital gyrus, including the lingual gyrus, is necessary for visual, verbal, and linguistic processing [61]. The temporal pole, which is unique to primates, has been implicated in high-order brain functions, such as mnemonic, language, semantic, visual, and socioemotional functions [62]. The temporal pole is considered to be a hub for information convergence and plays a role in AD [63]. In a study of aMCI patients, memory performance was inversely correlated with the temporal pole GM volume [64]. Finally, an rs-fMRI study found that the degree centrality value in the temporal lobe was higher in APOE ε4 carriers and was associated with enhanced cognitive performance [65]. Taken together, these studies provide neuroscientific and fMRI data supporting our findings. Our study helps bridge the gap between neuroscientific theory and clinical practice and may have far-reaching implications.

Our generalized linear model revealed that the global properties of the cortical-hippocampal network play a role in long-term delayed performance, which is a novel finding. More importantly, the FC value of the network modulated the relationships of long-term delayed recall with age. The prevalence of aMCI among rural-dwelling older adults in China was reported as 22.3%, and increased with age [66]. Age is a non-modifiable factor, which is closely associated with aMCI; nevertheless, our findings show that it may be possible to modulate the associations of age with long-term delayed recall performance.

We also found that local and global functions of the cortical-hippocampal network were closely related to general cognitive function, attention, executive function, and language. Our findings suggest that the cortical-hippocampal network could be targeted to improve cognitive function in one or multiple domains in aMCI patients. Close relationships of general cognitive function, executive function, and language with delayed recall have been reported in aMCI subjects [67]. In one study, aMCI subjects exhibited delayed recall impairments and performed worse on tests of attention [68]. Accumulating evidence from neuroimaging studies implicates prefrontal cortex dysfunction in the executive and attention impairments seen in aMCI [69].

### Limitations

This study also had several limitations. First, we did not take structural variations at the individual level into account. Second, inter-hemispheric effects on the cortical-hippocampal network were not analyzed due to the complexity of inter-hemispheric interactions and the limitations of DTI data. Third, since this was a symptom-based study, we did not check for signs of AD pathology in our aMCI subjects. Finally, prospective longitudinal studies are needed to identify the aMCI patients most likely to show progression to clinical dementia. We will aim to address these limitations in future research, and assess the curative potential of TMS in the context of our novel symptom-based brain network.

## Conclusion

In the present multimodal MRI study, we reported a novel symptom-based network, and the relationships of cognitive functions with brain function and structure. The cortex–hippocampus network constrained by the structural and functional connectome is closely related to long-term delayed recall.

## Supplementary Information

**Additional file 1: Supplemental Table 1.** Descriptions of global properties examined in the study.

## Data Availability

The data that support the findings of this study are available from the corresponding author upon reasonable request.

## Abbreviations

aMCI: Amnestic mild cognitive impairment
TMS: Transcranial magnetic stimulation
AD: Alzheimer’s disease
FC: Functional connectivity
MMSE: Mini-Mental State Examination
AVLT: Auditory Verbal Learning Test
MoCA-B: Montreal Cognitive Assessment-Basic
ACE-III: Addenbrooke’s Cognitive Examination III
BNT: Boston Naming Test
AFT: Animal Verbal Fluency Test
STT: Shape Trail Test
SDMT: Symbol Digit Modalities Test
DST: Digit Span Test
JLO: Judgment of Line Orientation
ECog: Everyday Cognition
PSQI: Pittsburgh Sleep Quality Index
TUGT: Timed Up and Go Test
HAMD: Hamilton Depression Scale
HAMA: Hamilton Anxiety Scale
