# Supplementary material for: Mapping the long-term delayed recall-based cortex-hippocampus network constrained by the structural and functional connectome: a case-control multimodal MRI study
Source: Alzheimers Res Ther. 2023 Mar 24;15:61. doi: 10.1186/s13195-023-01197-7 (PMC10037827; doi:10.1186/s13195-023-01197-7)
Supplement: Supplementary file 1 — Additional file 1: Supplemental Table 1. Descriptions of global properties examined in the study. [file 13195_2023_1197_MOESM1_ESM.docx]

**Supplemental Table 1:** Descriptions of global properties examined in the study

| **Parameter** | **Descriptions** |
| --- | --- |
| Assortativity | The tendency of nodes to link those nodes with similar number of edges |
| Hierarchy | identify the presence of a hierarchical organization in a network |
| Synchronization | How likely that all nodes fluctuate in the same wave pattern |
| Clustering coefficient (Cp) | The extent of local clustering of a network |
| Characteristic path length (Lp) | The extent of overall routing efficiency of a network |
| Sigma | The small-worldness indicating the extent of a network between randomness and order |
| Global efficiency (Eglobal) | How efficient of information propagation through the whole network |
| Local efficiency (Elocal) | How efficient of information propagation over a node's direct neighbors |

Note: All the global properties are only calculated in the cortical hippocampal network.

**Formula:**

**Assortativity**


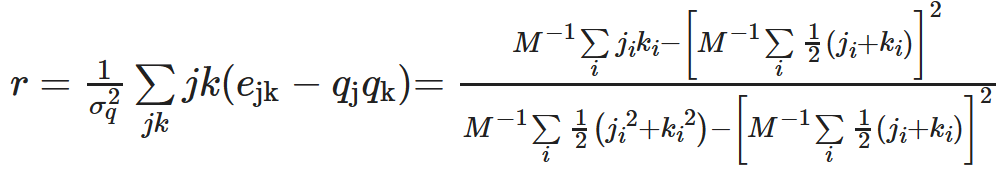


where e_jk_ was defined to be the joint probability distribution of the remaining degrees of the two vertices at either end of a randomly chosen edge, as well as σ^2^ _q_ was equal to the variance of q_k_/q_j._

**Hierarchy**

**
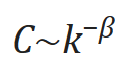
**

in which the *C* still represented the clustering and *k* indicated the degree of a node in a network. *β*, as the coefficient of hierarchy organization, was calculated fitting a linear regression with the ratio between log‐transformed *C* and log‐transformed *k*.

**Synchronization**

**
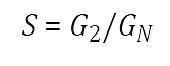
**

Synchronization is defined as the ration of the second smallest eigenvalue and the largest eigenvalue of the coupling matrix of network.

**Clustering coefficient (Cp)**

**
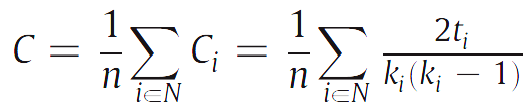
**

where *C_i_* is the clustering coefficient of node *i* (*C_i_ =* 0 for *k_i_* < 2).

**Characteristic path length (Lp)**

**
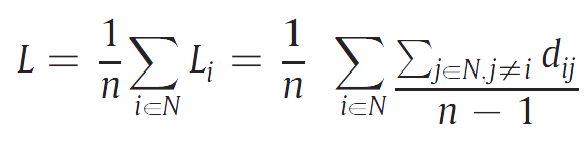
**

where *L_i_* is the average distance between node *i* and all other nodes.

**Sigma**

**
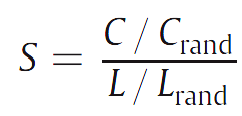
**

where *C* and *C*_rand_ are the clustering coefficients, and *L* and *L_rand_* are the characteristic path lengths of the respective tested network and a random network. Small-world networks often have *S* >> 1.

**Global efficiency (Eglobal)**

**
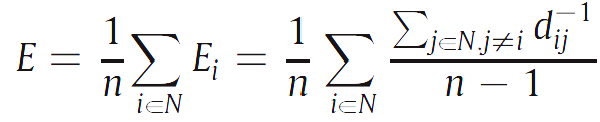
**

where *E_i_* is the efficiency of node *i.*

**Local efficiency (Elocal)**


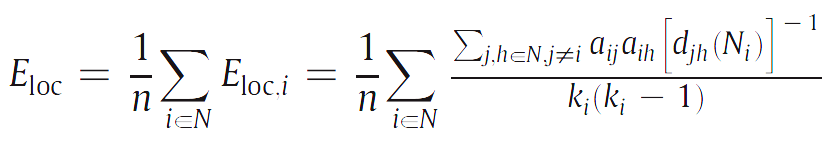


where *E_loc,i_* is the local efficiency of node *i*, and *d_jh_* (*N_i_*) is the length of the shortest path between *j* and *h*, that contains only neighbors of *i*.

**Reference:**

[1] Ravasz E, Barabási A L (2003): Hierarchical organization in complex networks. Phys. Rev. E. 67:026112.

[2] Rubinov M, Sporns O (2010): Complex network measures of brain connectivety: uses and interpretations. Neuroimage 52:1059 69.

[3] Wang JH, Zuo XN, Gohel S, Milham MP, Biswal BB, He Y (2011): Graph theoretical analysis of functional brain networks: test retest evaluation on short and long term resting state functional MRI data. PLoS One 6:e21976.
